# Real vs. immersive-virtual emotional experience: Analysis of psycho-physiological patterns in a free exploration of an art museum

**DOI:** 10.1371/journal.pone.0223881

**Published:** 2019-10-15

**Authors:** Javier Marín-Morales, Juan Luis Higuera-Trujillo, Alberto Greco, Jaime Guixeres, Carmen Llinares, Claudio Gentili, Enzo Pasquale Scilingo, Mariano Alcañiz, Gaetano Valenza

**Affiliations:** 1 Instituto de Investigación e Innovación en Bioingeniería, Universitat Politècnica de València, València, Spain; 2 Bioengineering and Robotics Research Centre E Piaggio & Department of Information Engineering, University of Pisa, Pisa, Italy; 3 Department of General Psychology, University of Padua, Padua, Italy; Victoria University of Wellington, NEW ZEALAND

## Abstract

Virtual reality is a powerful tool in human behaviour research. However, few studies compare its capacity to evoke the same emotional responses as in real scenarios. This study investigates psycho-physiological patterns evoked during the free exploration of an art museum and the museum virtualized through a 3D immersive virtual environment (IVE). An exploratory study involving 60 participants was performed, recording electroencephalographic and electrocardiographic signals using wearable devices. The real vs. virtual psychological comparison was performed using self-assessment emotional response tests, whereas the physiological comparison was performed through Support Vector Machine algorithms, endowed with an effective feature selection procedure for a set of state-of-the-art metrics quantifying cardiovascular and brain linear and nonlinear dynamics. We included an initial calibration phase, using standardized 2D and 360° emotional stimuli, to increase the accuracy of the model. The self-assessments of the physical and virtual museum support the use of IVEs in emotion research. The 2-class (high/low) system accuracy was 71.52% and 77.08% along the arousal and valence dimension, respectively, in the physical museum, and 75.00% and 71.08% in the virtual museum. The previously presented 360° stimuli contributed to increasing the accuracy in the virtual museum. Also, the real vs. virtual classifier accuracy was 95.27%, using only EEG mean phase coherency features, which demonstrates the high involvement of brain synchronization in emotional virtual reality processes. These findings provide an important contribution at a methodological level and to scientific knowledge, which will effectively guide future emotion elicitation and recognition systems using virtual reality.

## Introduction

The automatic quantification and recognition of human emotions is a research area known as "Affective Computing", which combines knowledge in the fields of psychophysiology, computer science, biomedical engineering and artificial intelligence [1]. Due to the central role that emotions play in many background processes, such as perception, decision-making, creativity, memory and social interaction, several studies have focused on trying to obtain a reliable methodology to evoke and automatically identify emotional states from objective psychometric measures [2]. Major exploitations of computational machines with affective intelligence focus on healthcare, education, marketing and entertainment [3,4], as well as on environmental psychology, i.e. the study of the effect of the environment on humans [5].

Irrespective of the application, two approaches have commonly been proposed to model emotions: discrete and dimensional models. The former proposes that there is a small set of basic emotions, assuming that complex emotions result from a combination of these basics, including anger, disgust, fear, joy, sadness and surprise [6]. Although discrete models are more easily understood by the non-expert, they are strongly criticized for lacking consistency and objective correlates (e.g. psychobiological and psychophysiological specific correlates) [7]. Dimensional models propose a multidimensional space where each dimension represents a fundamental property common to all emotions. The “Circumplex Model of Affect” (CMA) is one of the most used model, and refers to a Cartesian system of axes with two dimensions [8]: valence, i.e. the pleasantness or unpleasantness of an emotion; arousal, i.e. the intensity of the emotion in terms of activation from low to high.

To automatically classify emotions, correlates from, e.g. voice, face, posture, text, neuroimaging and physiological signals are widely employed [9]. In particular, several computational methods are based on variables associated with Central Nervous System (CNS) and Autonomic Nervous System (ANS) dynamics [9]. On the one hand, the use of the CNS to automatically classify emotion is justified by the fact that human emotional processing and perception involve activity of the cerebral cortex. In this regard, the electroencephalogram (EEG) is one of the techniques most used to measure CNS responses [10]. On the other hand, a wider class of affective computing studies exploits ANS changes on cardiovascular dynamics as elicited by specific emotional states, especially through Heart Rate Variability (HRV) analyses [11]. To this extent, recently proposed emotion recognition systems exploit wearable systems [12,13], allowing physiological monitoring in physical real-world environments through both HRV [14] and EEG [15].

Concerning the experimental emotional manipulation, the ability to reliably and ethically elicit affective states has proven to be a challenging task [16]. Based on the nature of the stimuli used to evoke emotional responses, two types are distinguished: active and passive. Active methods may involve behavioural manipulation [17], social psychological methods with social interaction [18], or dyadic interaction [19]. On the other hand, passive methods can fundamentally present images, sounds or films. Of note, regarding the images, the International Affective Picture System (IAPS) is one of the most prominent databases. It includes over a thousand depictions of people, objects and events standardized on the basis of valence and arousal [16]. IAPS has been used in many researches as an elicitation tool in emotion recognition methodologies [11].

Although many computational models have been successfully developed in lab environments using controlled stimuli, the influence of the level of immersion of the set-up (i.e. the objective description referring to the physical extent of the sensory information) has often been underestimated, thus eliciting emotional experiences not similar to real-world scenarios [20]. To overcome these limitations, researchers propose environmental-simulation technologies to replicate the experience of physical environments [21].

At present, Virtual Reality (VR) is one of the most powerful technologies that simulate experiences and provide the sensation of being in real situations [22]. In fact, 3D immersive virtual environments (IVE) have successfully been applied to phobias [23], presence [24], visualization technologies [25], quality of experience [26] and videogames [27]. Specifically, the main advantages of this technology are that: i) it allows us to isolate and modify variables in an efficient and low-cost way, something which is very difficult, or even impossible, in real environments [28]; and ii) it allows us to analyse an environment before its construction or environments far distant from the lab. Of note, VR can profitably be used to evoke emotions [28][29] and states of relaxation or anxiety [30]. Many VR researches have been performed using desktop or semi-immersive systems such as Powerwalls or caves [31]. Nowadays, the use of head-mounted displays (HMD) is growing due to their improved performance and decreased price. They are fully immersive devices that isolate the user from the external world. These devices, in fact, provoke a high sense of presence, understood as the illusion of "being-there" [32]. Note that HMDs have two main formats for displaying IVEs: 360° panoramas and 3D VR environments. 360° panoramas offer results closer to reality in terms of the participants' psychological responses, while 3D VR in terms of their physiological responses [33]. In addition, 3D VR allows the user to freely interact with the environment.

The comparison between the responses evoked by physical environments and their virtual simulations has been studied to some degree through the assessment of psychological responses [34], cognitive performance [35] and—to a much lesser extent–physiological and behavioural responses [36][37]. Although differences have been found, environmental simulations achieve a considerable level of general validity [38]. However, in the variety of studies undertaken, simulations have not yet been comprehensively compared with the real world in the analysis of emotional experiences, especially by employing a thorough analysis of CNS and ANS dynamics.

To this end, the main aim of the present study is to perform an exploratory research to comparatively and quantitatively investigate the psychological and physiological patterns evoked during, first, free exploration of a real art museum and, second, where they visualize a virtualization of the museum through a 3D IVE.

To this extent, firstly, we undertake a psychological comparison, using self-assessment tests, for both real and virtual environments. Secondly, we perform a comprehensive physiological comparison using brain and cardiovascular linear and nonlinear dynamics to build arousal and valence-specific classifiers. Thirdly, we analyse the inclusion of 2D (i.e. IAPS images) and 360° standardized emotional stimuli as a part of the calibration phase of the classifier. Moreover, at an exploratory level, we also investigate differences and similarities in psychophysiological responses elicited by real and virtual environments. To this end, we develop emotion recognition models for real vs. immersive-virtual scenario comparison to determine if the subject is experiencing a virtual or real emotional experience. Classification accuracies are gathered from nonlinear Support Vector Machine algorithms and a set of EEG and HRV features extracted using various state-of-the-art metrics. Methodological details, the experimental results, and the discussion and conclusion follow below.

## Materials and methods

### Experimental design

**Fig 1:**
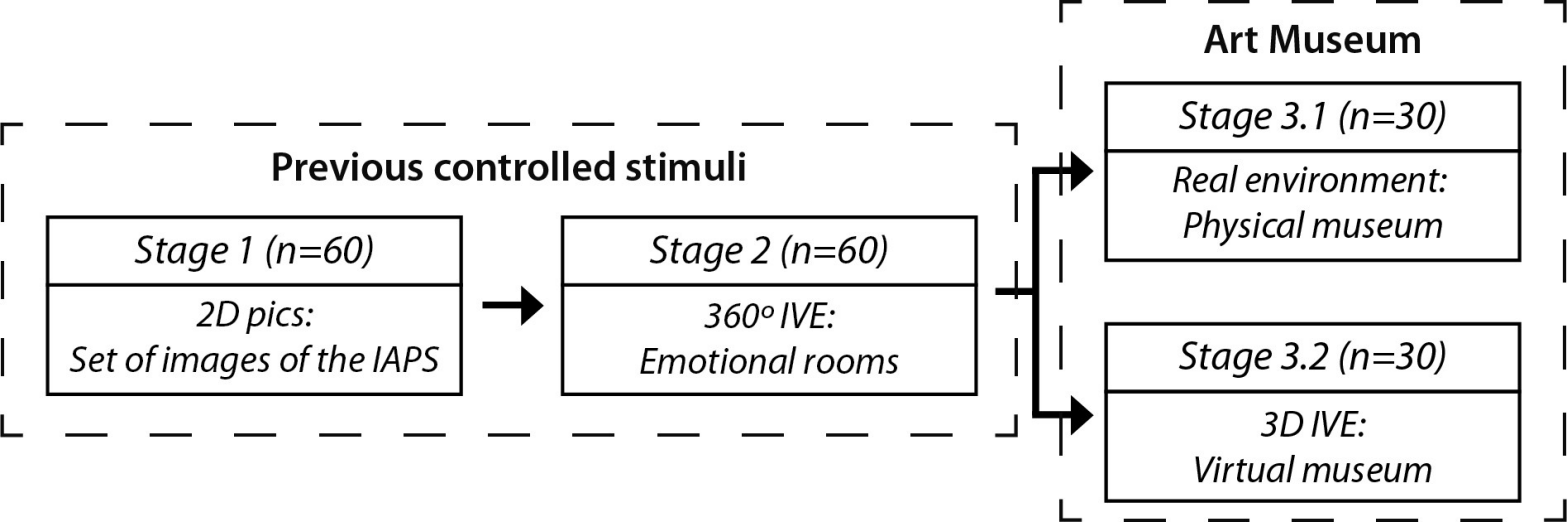
Experimental phases of the research. n represents the number of subjects involved in each stage.

An exploratory study was conducted in two different phases, including two prior stages using controlled stimuli. Each stage was presented consequently (Fig 1), with signal acquisition independently recorded. Between each stage, the subjects rested for 3 minutes, sitting on a chair. Stage 1 consisted of showing the subjects 2D pictures based on IAPS. Stage 2 consisted of a 360° panorama emotion IVE presented in an HMD. Finally, the last stage in both phases consisted of the free exploration of a museum exhibition. However, in Stage 3.1, the subjects explored a real museum exhibition and in Stage 3.2 the subjects explored the 3D virtual reality simulation of the same exhibition. Each subject was randomly assigned to undergo either Stage 3.1 or Stage 3.2.

The ethics committee of the Polytechnic University of Valencia approved the experimental protocol. All methods and experimental protocols were performed in accordance with the guidelines and regulations of the local ethics committee of the Polytechnic University of Valencia. Written informed consent was obtained from all participants involved in the experiment. In particular, the individual in this manuscript has given written informed consent (as outlined in the PLOS consent form) to publish these case details.

### Stimulus elicitation

#### Previous controlled stimuli

In *Stage 1* we developed an affective elicitation using standardized 2D pictures. This was achieved by projecting a set of images onto a monitor (Dell E198FPb, LCD, 19-inch, 1280x1024 @ 75Hz). At first, the users were asked to rest for 4 minutes while looking at a blank image (B), in order to start the exploratory study from a relaxed status. This period was divided into: one open eye minute; one closed eye minute; one open eye minute; and one closed eye minute. Thereafter, the affective elicitation began. We took inspiration from the elicitation methodologies reported in previous works [10,11], with minor changes. Briefly, the slideshow comprised of 9 image sessions, alternating neutral sessions (from N1 to N5) and arousal sessions (from A1 to A4). The order of presentation of the images was random. One-minute resting-state sessions (from R1 to R8) were placed between each neutral/arousal session. Each arousal session was divided into 3 blocks of valence (from V1 to V3). Thus, 1 block of neutral pictures and 12 blocks of non-neutral pictures were displayed. Further details are reported in the supporting information. The overall protocol used 110 images. Each image was presented for 10 seconds for the whole duration of the experiment, 18 minutes and 20 seconds.

**Fig 2:**
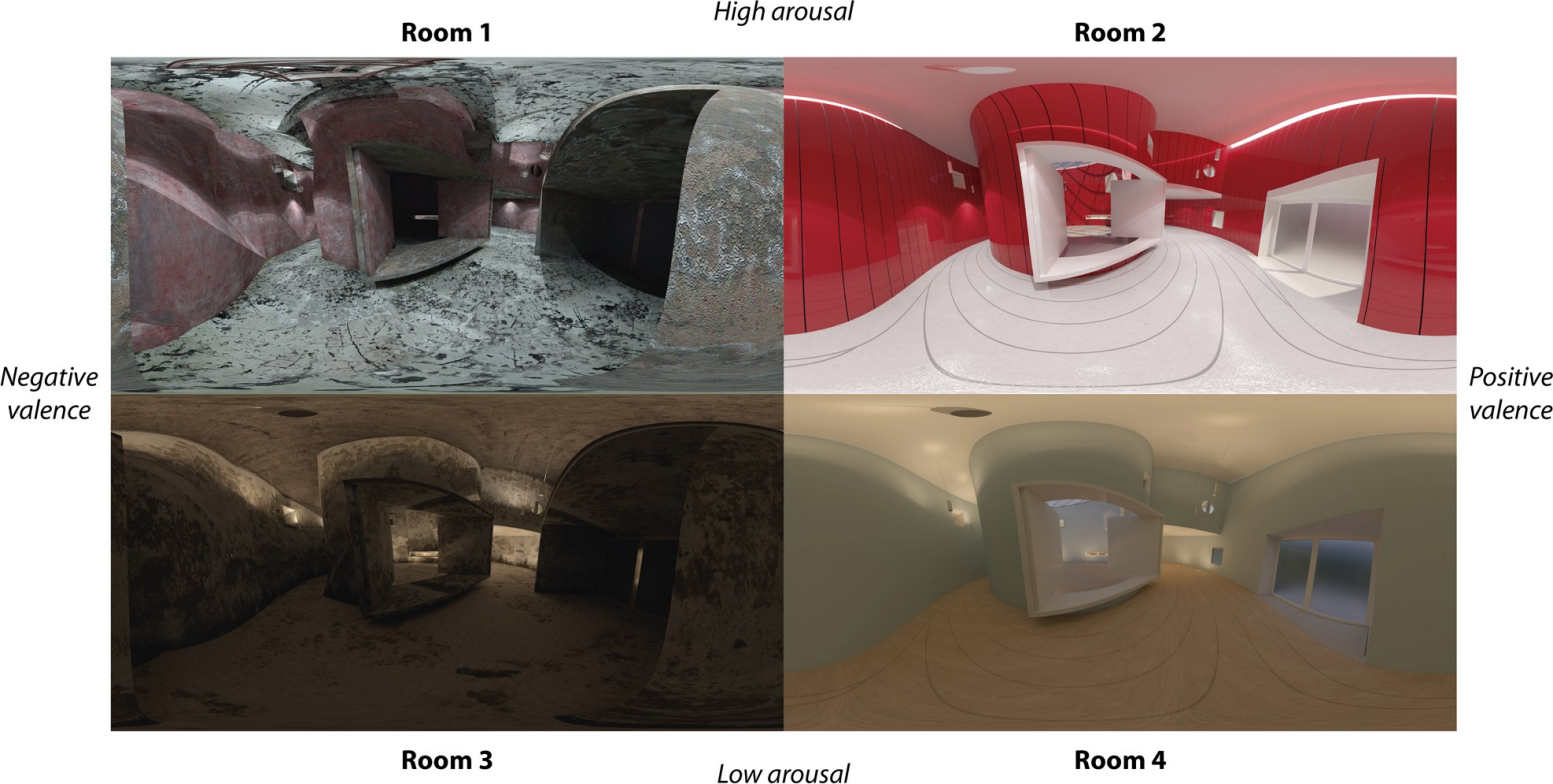
360° panoramas used in stage 2.

In *Stage 2*, we developed an affective elicitation using architectural environments displayed by 360° panoramas implemented in a portable HMD. The stimuli had been analysed and validated in previous research [13]. This type of environment was chosen as the influence of architectural environments on affective-behavioural responses is widely accepted [39]. Previous research shows that subtle variations in the space may generate different neurophysiological responses [40]. In addition, previous works show that the 360° panorama-format using HMD devices is a valid set-up for evoking psychological and physiological responses similar to those that physical environments evoke [25]. Hence, four architectural environments were proposed as representative of four emotional states (Fig 2.), following the CMA [41]. The emotional rooms were designed based on different variations of the same base-scenario, “Villa in the forest”, by Kazuyo Sejima [42]. The research team, which included architects, considered this an appropriate base from which to make modifications to generate different moods. The architectural parameters used to modify the base-scenario were illumination, colour and geometry.

**Fig 3:**
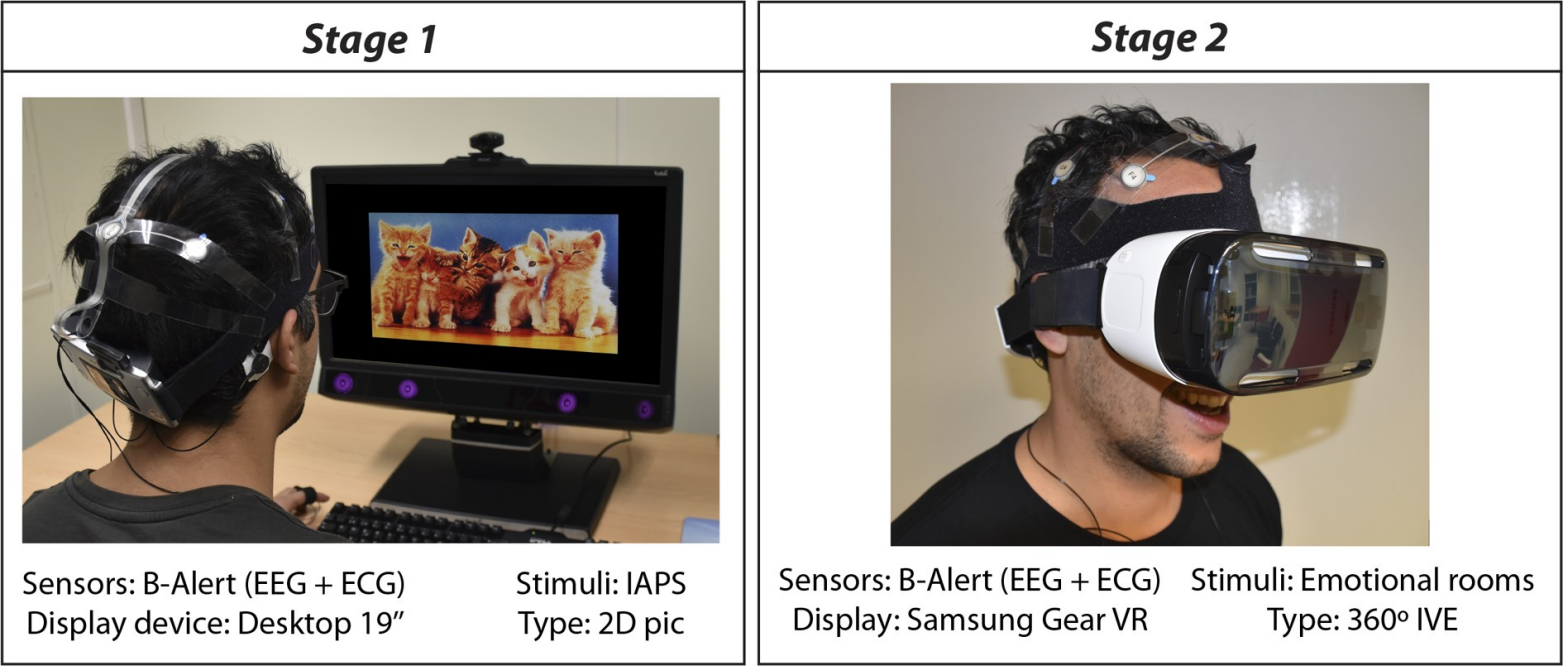
Example of experimental set-up of stage 1 and 2.

Technically, the process of developing the four architectural environments consisted of modelling and rendering. Modelling was performed by using Rhinoceros v5.0 (www.rhino3d.com) and rendering was performed using the VRay engine v3.00.08 (www.vray.com), operating from Autodesk 3ds Max v2015 (www.autodesk.es). Renders were exported in .jpg format with resolutions of 6000x3000 pixels at 300 dots per inch. The 360° panoramas were implemented in Samsung Gear VR HMDs and the reproduction was fluid and uninterrupted. The Samsung HMD has a stereoscopic screen of 1280×1440 pixels per eye and a 96° field of view, supported by a Samsung Note 4 mobile telephone with a 2.7GHz quad-core processor and 3GB of RAM. Fig 3 shows an example of experimental set-up of Stages 1 and 2.

Regarding the protocol, each room was presented for 1.5 minutes and the sequence was counter-balanced using the Latin Square method. After viewing each room, the users were asked to orally self-assess the emotional state evoked by each room using a SAM questionnaire embedded in the 360° photo, ranging from -4 to 4, for arousal and valence dimensions.

#### Physical museum exhibition

**Fig 4:**
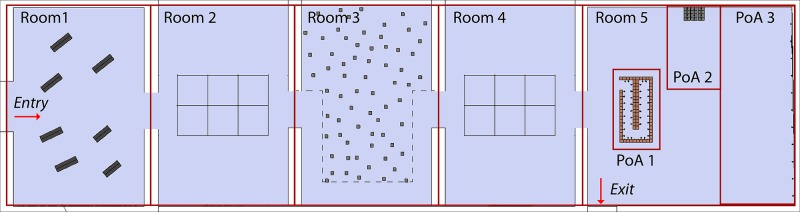
Plan of the art-exhibition with the 5 rooms and 3 pieces of art.

In *Stage 3*.*1*, we performed an affective elicitation using a physical environment. An art exhibition was chosen in order to evoke an intense emotional experience. The Institut Valencià d'Art Modern (IVAM) offered us their facilities to undertake our study. We selected the art-exhibition “Départ-Arrivée”, by Christian Boltanski, because it had a very emotional topic, the Nazi Holocaust. The exhibition had 5 rooms and an area of approximately 750 m^2^ (Fig 4).

The subjects were asked to explore freely the first four rooms. When they entered the fifth room, they had also to explore it freely, but they were, in addition, required to stop and study the three pieces of art in detail. The researcher waited for the subject at the exit door, allowing the subject to freely explore the exhibition.

**Fig 5:**
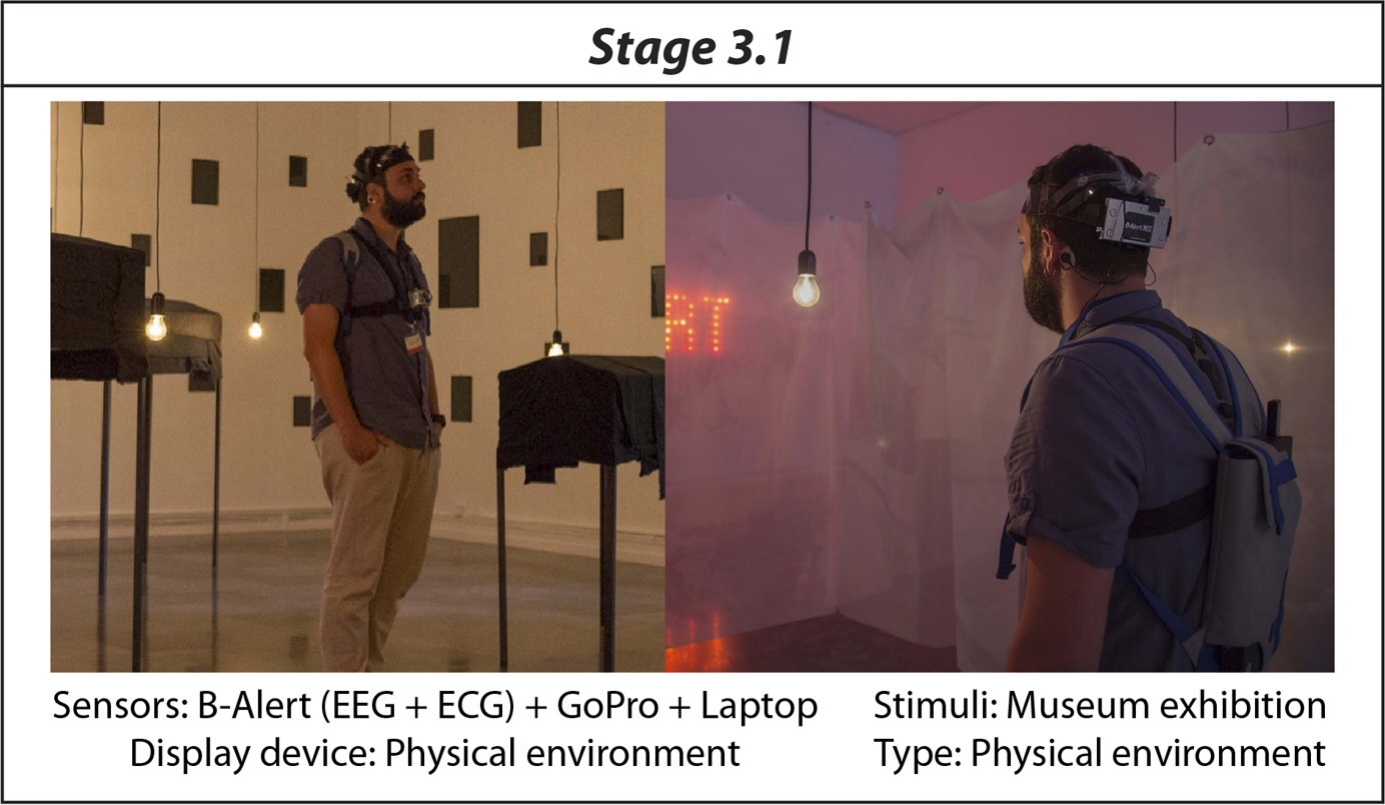
Example of experimental set-up of Stage 3.1.

In order to track the position of the subjects, therefore being sure that she/he visited all rooms, we used a GoPro camera, that subjects carried attached to their chests by means of a suitable harness. The physiological signals were recording on a laptop that the subject carried in a backpack. Fig 5 shows an exemplary experimental set-up of Stage 3.1.

After the museum exploration, the subjects were asked to complete two questionnaires. In the first, they evaluated the emotional impact of each of the five rooms and the three pieces of art, using a SAM questionnaire and a photo of each room. In the second, we presented two questions to evaluate the subjective impact of the sensors: (1) “During the test, did you feel annoyed by the sensors?”; (2) “During the test, was there ever a time when you forgot that you were sensorized?”. The subjects who reported feeling “moderately” or “a lot” annoyed were excluded from further analyses.

#### Virtual museum exhibition

**Fig 6:**
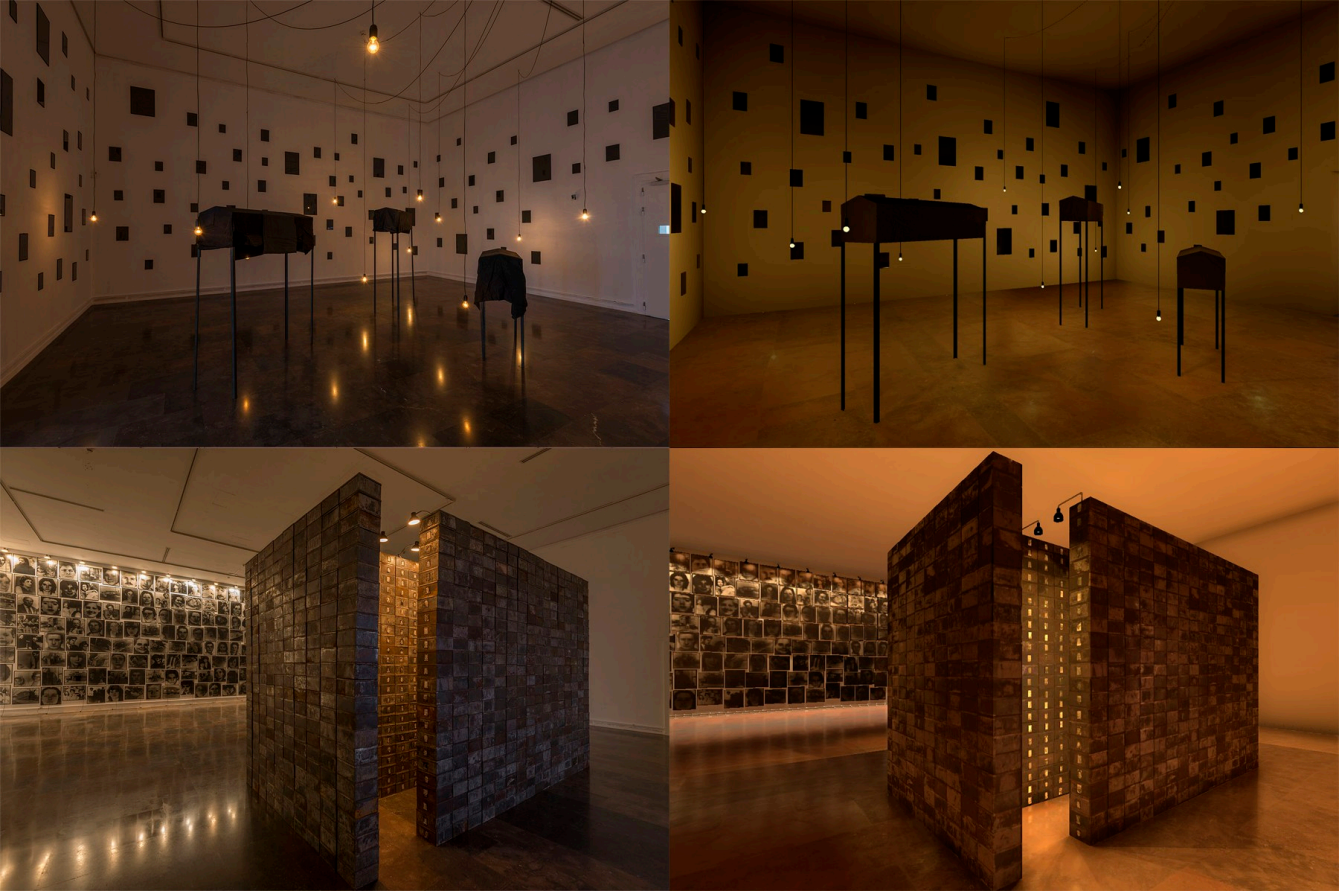
**Comparison between the physical museum (left) and the virtual museum (right).** The photos represent Room 1 and Room 5.

**Fig 7:**
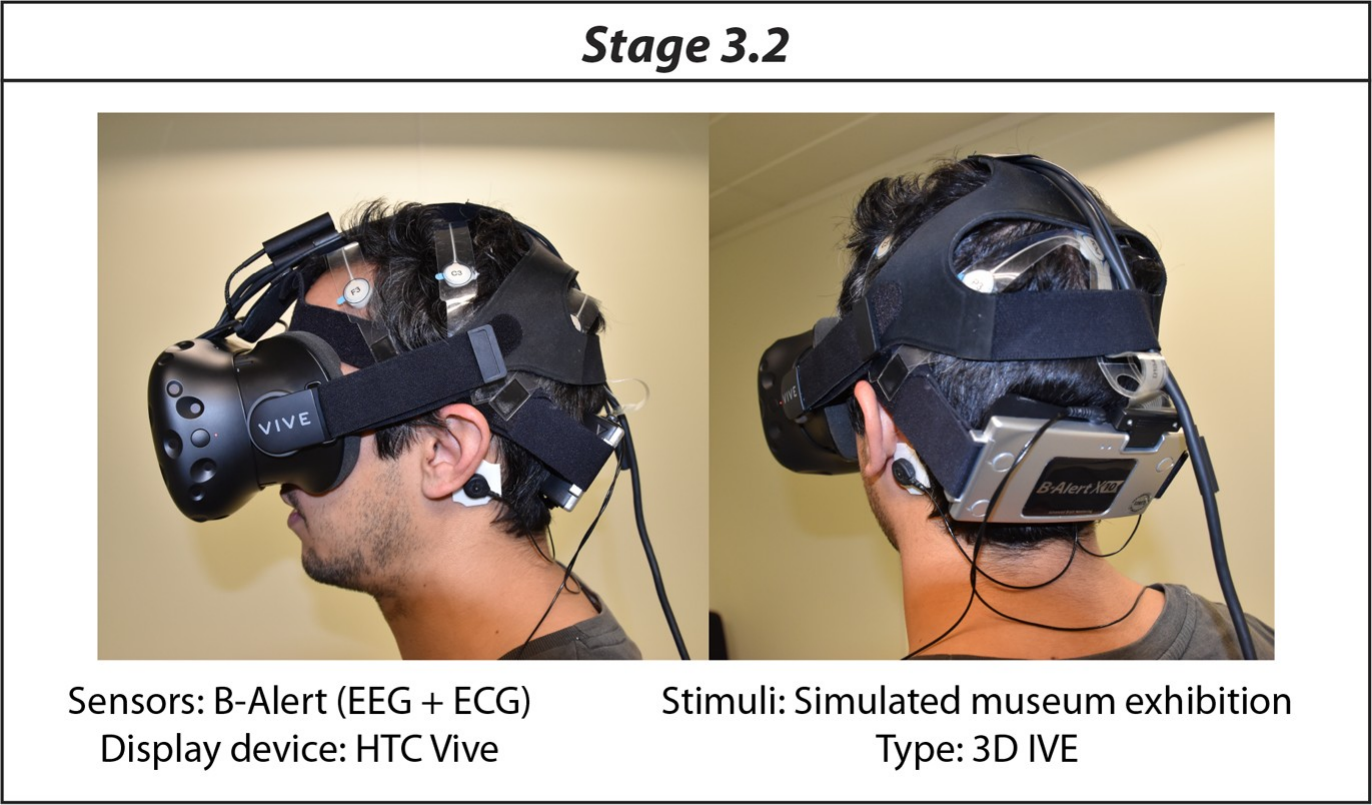
Exemplary experimental setup of Stage 3.2.

In *Stage 3*.*2*, an affective elicitation was performed through the 3D VR representation of the museum exhibition visited in phase 1. The Unity 5.1 game engine (www.unity3d.com) was used. A three-dimensional representation of the museum exhibition was provided by Rhinoceros v5.0. Textures partially extracted from the physical environment were imported to achieve maximum realism. This involved exhaustively and methodologically drawing and photographing the entire exhibition. Exemplary photographs of the real environment and screenshots of the virtual environment are shown in Fig 6. Further examples are in the supporting information. Regarding the 3D VR simulation, the developed scenario was compiled for HTC Vive (www.vive.com). This system allows visual and displacement simulations. On the one hand, visualization is performed using an HMD with 2160x1200 pixels (1080×1200 per eye) and a field of view of 110 degrees working at 90Hz refresh rate. On the other hand, displacements are performed using tracking technology, two controllers and two base stations that, together, allow the subject to interact with their environment and physically move within an area of a 2x2 metres. Specifically, the teleport navigation metaphor included in the HTC Vive developed tools was used, with a 2.5 metres from the subject maximum teleportation radio. It was chosen in order to achieve pseudo-naturalistic navigation, allowing the subjects to take large steps. The entire system was connected to the research PC (Predator G6, www.acer.com) via DisplayPort 1.2 and USB 3.0, running smoothly and without interruptions. Fig 7 shows an exemplary experimental set-up of Stage 3.2.

Before starting this stage of the experiment, the subjects were placed in a neutral scenario, which displayed only a floor, without any texture. A screenshot of this scenario is included in the supporting information. They were asked to undertake a period of training in this place. They could take all the time that they needed inside this scenario, until they considered their adaptation to VR and the navigation metaphor complete. After this, the instructions for the virtual museum exhibition were exactly the same as for the physical exhibition. The subject´s navigation was also displayed in real time on a desktop and the researcher used this to note when the subject arrived at the exit, in order to stop the recording and remove the HMD.

Following the exploration of the virtual museum, the subjects were asked to answer the same two questionnaires as for Stage 3.1: (1) affective self-assessment evaluation of the rooms and pieces of art; (2) impact of the sensors in the behaviour of the subjects. In addition, in this phase the subjects had to answer a questionnaire about presence in the virtual museum. We used the well-known “SUS questionnaire” [43]. Its current version consists of six items, rated on 1-to-7 Likert scale, measuring three aspects of the subject’s senses: the experience of being inside the simulation; the consideration of the simulation as the dominant reality; and the memory of the simulation as a place.

### Participants’ eligibility and group homogeneity

A homogeneous population of 60 healthy subjects (age 28.9 ± 5.44, 40% male, 60% female), suffering neither from cardiovascular nor obvious mental pathologies, was recruited to participate in the exploratory study. They were divided into 30 subjects for the first phase and 30 for the second. The following were the criteria to participate in the study: age between 20 and 40 years; Spanish nationality; not having formal education in art or a fine-art background; not having any previous virtual reality experience; and not having previously visited this particular art exhibition.

Two questionnaires were included to ensure that the subjects were in a healthy mental state and constituted a homogeneous group. In the first, all participants were screened by a Patient Health Questionnaire (PHQ) [44]. Only participants with a score lower than 5 were included in the study to avoid the presence of either middling or severe mental disorders. In the second, a self-assessment, based on a selection of IAPS pictures [45] using the Self-Assessment Manikin (SAM) [46], was administered. The presented set consisted of different degrees of arousal and valence perception (arousal from 3.41 to 7.24; valence from 1.29 to 8.17; pictures selected: 7234, 5201, 9290, 1463, 9181, 8380, 3102, 4652).

The self-assessment values were used to analyse if any subject had an emotional response that could be considered as an outlier with respect to standard elicitations. To this end, the arousal and valence of each subject were standardized through a z-score using the mean and deviation of the IAPS published scores [45]. Standardized evaluations outside of the range -2.58 to 2.58 (i.e., α = 0.01) were designated as outliers [47]. Subjects with outliers were excluded from further analyses, while we retained the emotional responses that belong statistically to 99% of the population as published in the IAPS. In addition, the subjects whose signal recording experienced errors were rejected, e.g. because of disconnection of the sensors during the elicitation. The participants had successfully to complete all the stages.

### Physiological signals and instrumentation set

The electroencephalographic (EEG) and electro-cardiographic (ECG) signals were acquired using B-Alert x10 (Advanced Brain Monitoring, Inc. USA). This provides an integrated approach for wireless wearable acquisition, sampled at 256 Hz. Regarding the EEG, the location of the sensors was in the frontal (Fz, F3 and F4), central (Cz, C3 and C4) and parietal (POz, P3, and P4) regions based on international 10–20 electrode placement. A pair of electrodes placed below the mastoid was used as a reference. A test was performed to check that the impedances of the electrodes were below 20kΩ. In order to check the proper conductivity of the electrodes, a test was performed. Concerning the ECG, the left lead was located on the lowest rib and the right lead on the right collarbone. Data from 15 subjects out of 60 were rejected due to poor quality.

### Signal processing

Firstly, the signals were synchronized and segmented for each stage. The methodology used is detailed in the supporting information. Then, HRV and EEG signal processing methods were applied to extract features to characterize the physiological responses to the stimuli.

#### Heart rate variability

To obtain the RR series from the ECG, we implemented the Pan-Tompkins’s algorithm for QRS complex detection. The individual trends components were removed using the smoothness prior detrending method [48]. Artefacts and ectopic beats were corrected through the use of Kubios HRV software [49]. From the RR series, we performed the analysis of the standard HRV parameters in the time and frequency domains. In addition, we included other HRV measures quantifying heartbeat nonlinear and complex dynamics [50]. Table 1 presents a list of features included.

**Table 1:** List of HRV features used.

Time domain	Frequency domain	Other
Mean RR	VLF peak	Poincaré SD1
Std RR	LF peak	Poincaré SD2
RMSSD	HF peak	Approximate Entropy (ApEn)
pNN50	VLF power	Sample Entropy (SampEn)
RR triangular index	VLF power %	DFA α1
TINN	LF power	DFA α2
	LF power %	Correlation dimension (D2)
	LF power n.u.	
	HF power	
	HF power %	
	HF power n.u.	
	LF/HF power	
	Total power	

The time domain analysis includes the following features: average and standard deviation of the RR intervals, the root mean square of successive differences of intervals (RMSSD), the number of successive differences of intervals which differ by more than 50 ms (pNN50), the triangular interpolation of the HRV histogram and the baseline width of the RR histogram evaluated through triangular interpolation (TINN). The features of the frequency domain were calculated using a power spectrum density (PSD), applying Fast Fourier Transform. The analysis was performed in three bands: VLF (very low frequency, <0.04 Hz), LF (low frequency, 0.04–0.15 Hz) and HF (high frequency, 0.12–0.4 Hz).

For each of the three frequency bands we calculated the peak value (corresponding to the frequency having maximum magnitude) and the power of each frequency band in absolute and percentage terms. Normalized power (n.u.) was calculated for the LF and HF bands as the percentage of total power, subtracting previously the power of VLF to the total power. The LF/HF ratio was calculated to quantify sympatho-vagal balance and to reflect sympathetic modulations [50]. Moreover, the total power was calculated.

Finally, many features were extracted using nonlinear analysis, as they were shown to be important quantifiers of cardiovascular control dynamics mediated by the ANS in affective computing [11]. Firstly, Poincaré plot analysis was applied. It is a quantitative-visual technique, whereby the shape of a plot is categorized into functional classes, providing summary information of the behaviour of the heart. SD1 is associated with fast beat-to-beat variability and SD2 analyses the longer-term variability of R–R [50]. An entropy analysis was included, using Sample Entropy (SampEn) and Approximate Entropy (ApEn). SampEn provides an evaluation of time-series regularity [51] and ApEn detects changes in underlying episodic behaviour not reflected in peak occurrences or amplitudes [52]. DFA correlations analyse short-term and long-term fluctuations through the α1 and α2 features, where α1 represents the fluctuation in range of 4–16 samples and α2 refers to the range of 16–64 samples [53]. Finally, the D2 feature measures the complexity or strangeness of the time series. This is expected to provide information on the minimum number of dynamic variables needed to model the underlying system [54].

#### Electroencephalographic signals

**Fig 8:**
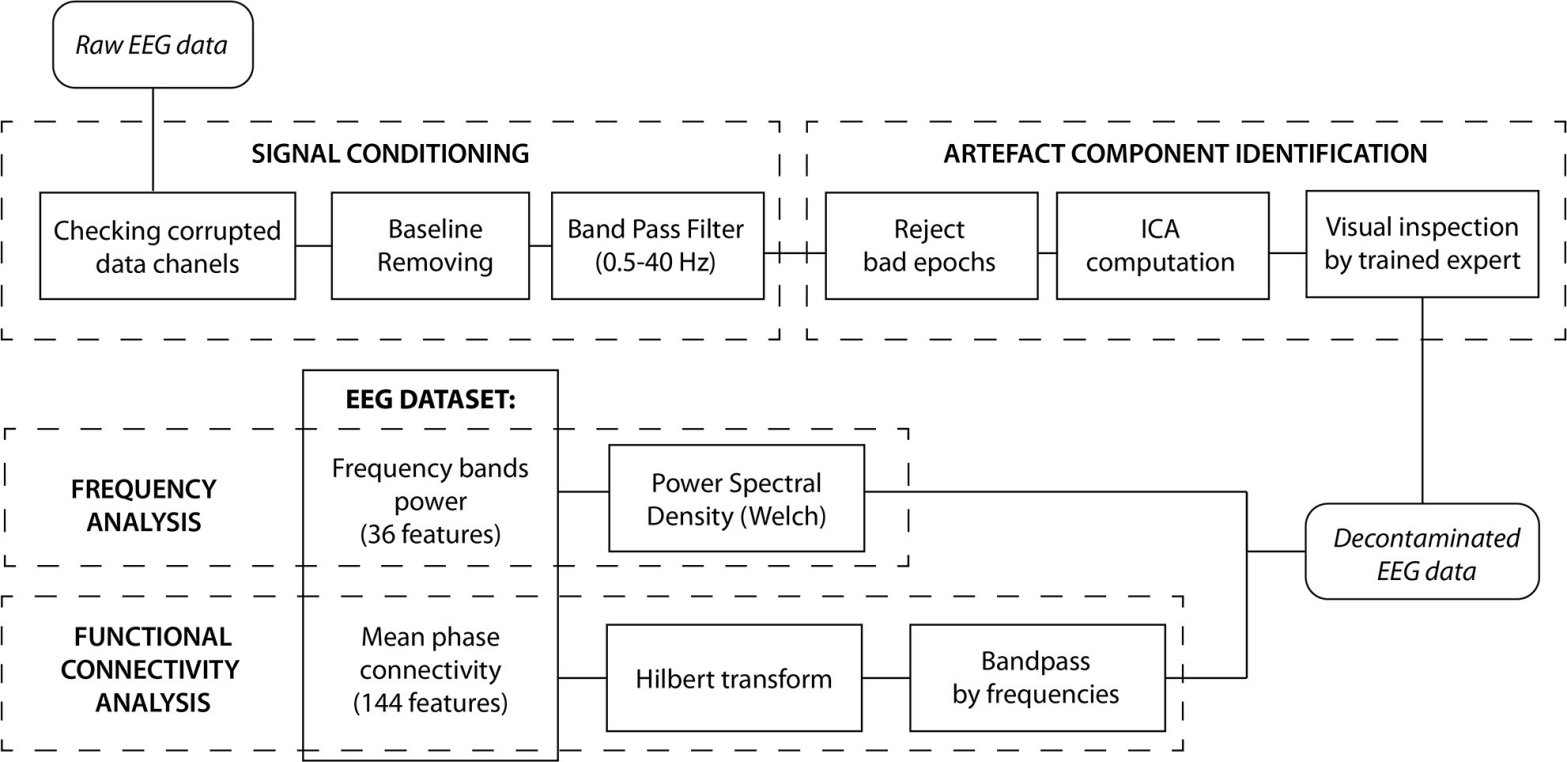
Block scheme of the EEG signal processing steps.

Fig 8 shows the complete EEG processing scheme, which is performed using the open source toolbox EEGLAB [55].

Firstly, the data from each channel was analysed to identify corrupted channels using the fourth standardized moment (kurtosis) along the signal of each electrode [56]. Moreover, the channel was also classified as corrupted if the signal was flatter than 10% of the total stage duration. If a channel was considered as corrupted, it could be interpolated from its neighbouring electrodes. The subject would be rejected if more than one channel was corrupted. Among all of the subjects, only one channel was interpolated.

The EEG baseline was removed by mean subtraction and a band pass filter between 0.5 and 40 Hz. The signal was segmented in epochs of one-second duration. Moreover, an automatic artefact detection was applied, rejecting epochs when more than 2 channels contained samples which exceeded an absolute threshold of 100.00 μV and a gradient of 70.00 μV between samples [57]. The Independent Component Analysis (ICA) [58] with an infomax algorithm was performed to identify and remove components due to blinks, eye movements and muscular artefacts. The components were analyzed by a trained expert to identify and reject those related to artefacts. The effectiveness of the algorithms used to detect and remove artefacts was carefully checked by visual inspection. The subjects who had more than one third of their signals affected by artefacts were rejected. Spectral and functional connectivity analyses were performed after the pre-processing.

An EEG spectral analysis was performed to estimate the power spectra in each epoch, within the frequency bandwidth: θ (4–8 Hz), α (8–12 Hz), β (13–25 Hz), γ (25–40 Hz). Frequency band δ (< 4Hz) was not taken into account in this study. It was performed using Welch’s method with 50% overlapping. 36 features were obtained from the 9 channels and 4 bands. The functional connectivity analysis was performed using Mean Phase Coherence [59]. It was performed for each pair of channels in each band:

$$R^{2}=E{\left[cos(\Delta \phi )\right]}^{2}+E{\left[sin(\Delta \phi )\right]}^{2}$$

Where *R* is the MPC, Δϕ is the relative phase difference between two channels derived by the instantaneous difference of the analytics signals from the Hilbert transform, and *E* is the expectation operator. MPC values can oscillate between 0 and 1. The MPC is close to 1 when a strong phase synchronization exists between two channels. Alternatively, MPC is close to 0 if the two channels are not synchronized. From each combination of a pair of 9 channels in one specific band, 36 features were extracted. Consequently, 144 features were developed from the 4 bands analysed.

### Data fusion and pattern recognition

**Fig 9:**
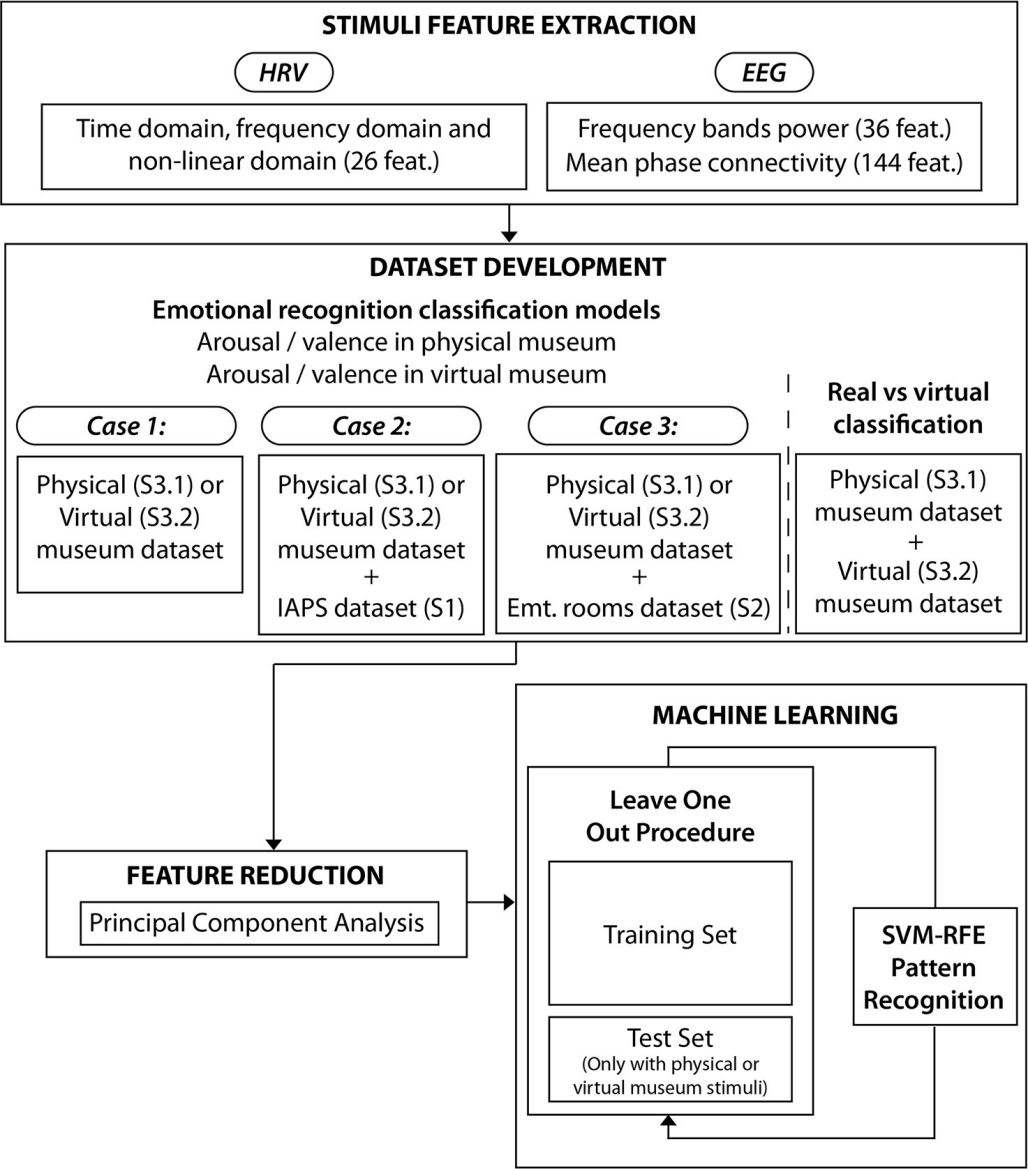
Overview of the data analysis and emotional pattern recognition.

An overview of the emotion recognition classification scheme is shown at Fig 9. For each stimulus, HRV features were calculated using the time windows defined in the segmentation methods. Concerning EEG, we considered the mean of the time-windows of the stimuli as the representative value in both analyses. Therefore, each stimulus (pictures, emotional rooms and museum rooms/pieces of art) was described by 209 features.

Four classification models were independently developed: arousal level in the physical museum, valence level in the physical museum, arousal level in the virtual museum, and valence level in the virtual museum.

For each model, the datasets of stimuli were created using three analytical cases: (1) using only the museum data, (2) including IAPS data and (3) also including the 360° data. The different cases were performed to test the following analyses.

*Case 1*: *Physical or Virtual Museum*. The features of stimuli from Stage 3.1 were used in order to analyse emotion recognition in the physical museum. In addition, the features of the stimuli from Stage 3.2 were independently used to analyse emotion recognition in the virtual museum.

*Case 2*: *Physical or Virtual Museum + IAPS*. In order to analyse the influence of including standardized 2D image responses in the emotional models, the features of Stage 1 stimuli were concatenated with the physical museum feature stimuli (Stage 3.1) or virtual museum feature stimuli (Stage 3.2).

*Case 3*: *Physical or Virtual Museum + Emotional Rooms*. In order to analyse the influence of including 360° IVE responses in the models, the features of Stage 2 stimuli were concatenated with the physical museum feature stimuli (Stage 3.1) or virtual museum feature stimuli (Stage 3.2).

In each emotional model, the class label was bipolarized into high/positive (>0) and low/negative (< = 0) for both arousal/valence.

Finally, a 2-class pattern recognition algorithm discerning between real vs. virtual museum exploration was developed, i.e. a classifier that aims to recognize if the emotional experience is elicited from a virtual or real scenario.

In all the classification models, including the emotional and real vs. virtual classifier, a feature reduction strategy was adopted to decrease the dimension of the dataset due to the high-dimensional feature space obtained. We implemented the Principal Component Analysis method (PCA) [60], which is based on the linear transformation of the different variables in the principal components. We included the features that explained 95% of the variability of the dataset. The PCA was independently applied in the three analyses. In order to validate the machine learning models, the Leave-One-Subject-Out (LOSO) cross-validation procedure was applied, using Support Vector Machine (SVM)-based pattern recognition [61]. For the LOSO scheme, the training set was normalized by subtracting the median value and dividing by the median absolute deviation over each dimension.

In each iteration, the validation set consisted of the stimuli of the physical or virtual museums of one specific subject; it was normalized using the median and deviation of the training set. Regarding the learning model, a C-SVM with sigmoid kernel function was used. The parameters of cost and gamma were optimized using a vector with 15 parameters logarithmically spaced between 0.1 and 1000. Moreover, we performed a feature selection strategy to explore the relative importance of each feature. A support vector machine recursive feature elimination (SVM-RFE) procedure, in a wrapper approach, was included. It was performed on the training set of each fold and we computed the median rank for each feature over all folds.

We specifically chose a recently developed nonlinear SVM-RFE which includes a correlation bias reduction strategy in the feature elimination procedure [62]. The model was optimized to achieve best accuracy whenever it has a balanced confusion matrix. We consider a model balanced when its confusion matrix has a true positive and a true negative over 60%. The algorithms were implemented using Matlab© R2016a and LIBSVM toolbox [63].

## Results

### Subjects’ self-assessment

No subjects showed depressive symptoms according to their PHQ-9 scores. The mean and standard deviations of the PHQ-9 questionnaires were 3.31 ± 2.57. Considering the IAPS self-assessment, a total of 8 subjects were considered outliers with respect to standard emotion elicitations.

Regarding the Stage 2, the evaluation of the subjects for each IVE averaged using mean and standard deviation in terms of arousal were (Room 1: 1.17 ± 1.81, Room 2: 2.10 ± 1.59, Room 3: 0.05 ± 2.01, Room 4: -0.60 ± 2.11) and valence (Room 1: -1.12 ± 1.95, Room 2: 1.45 ± 1.93, Room 3: -0.40 ± 2.14, Room 4: 2.57 ± 1.42), achieving the emotion statement for which they were designed, except in the case of arousal in Room 3.

**Fig 10:**
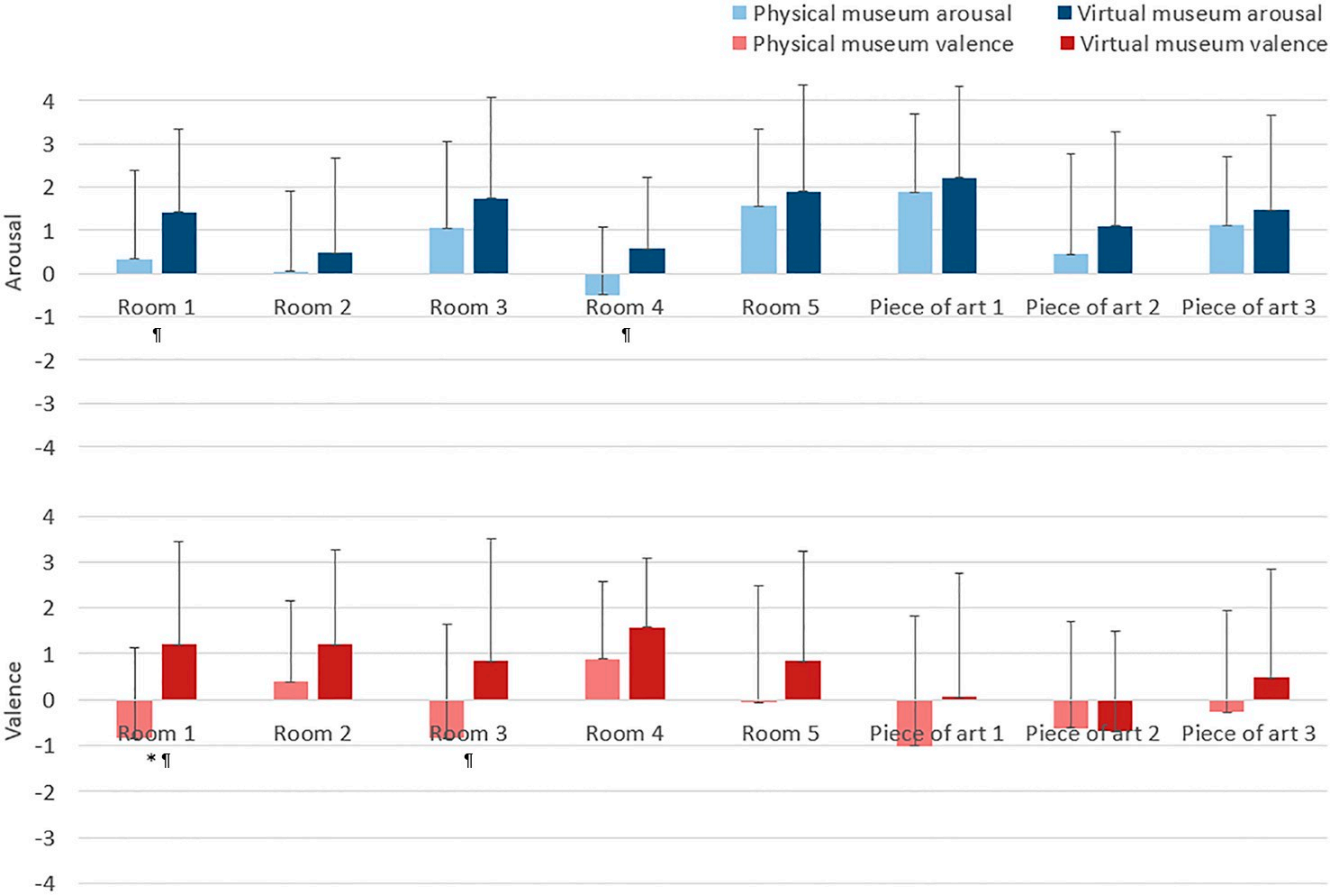
Self-assessment scores in physical and virtual museums using SAM and a Likert scale between -4 and +4. Bars represent the means, vertical lines represent the standard deviation of the means, blue represents arousal, and red valence. (* indicates significant differences with p < 0.05, ^¶^ indicates significant differences with p < 0.1).

Concerning Stages 3.1 and 3.2, Fig 10 shows the self-assessment of the subjects for the museum stimuli (rooms and pieces of art), using mean and standard deviations in terms of arousal and valence. Due to the non-Gaussianity of data (p < 0.05 from the Shapiro-Wilk test with null hypothesis of having a Gaussian sample), the Mann-Whitney U test was applied (α<0.05). Along the arousal dimension, no significant differences were found. Regarding valence, only room 1 showed a significant difference (p-value = 0.006). In addition, we analyse the stimuli considering a second alpha threshold (α<0.1) in order to decrease the probability of perform a type II error. In this case, room 1 (p-value = 0.084) and room 4 (p-value = 0.053) show higher arousal in virtual condition, and room 1 (p-value = 0.006) and room 3 (p-value = 0.051) present higher valence in virtual condition. After the bipolarization of scores (positive/high >0), the physical museum presents 59.72% of high arousal and 40.97% of positive valence values; and the virtual museum presents 71.71% of high arousal and 61.84% of positive valence values.

### Emotion recognition classification

Table 2 shows an overview of the results of the four emotion recognition models in three analysis cases. Regarding arousal recognition in the physical museum, not including IAPS or emotional rooms data, the accuracy is 68.05%. In the case where IAPS and emotional rooms data were included, the accuracy increases by 3.47%, reaching 71.52% in both cases. In all cases the model used features of the three analyses and the confusion matrices were balanced.

**Table 2:** Level of emotion recognition.

Analysis cases	Feature	Accuracy	F-Score	ΔAccuracy	Confusion matrix				Featured used			
					True high/pos	False high/pos	False low/neg	True low/neg	Total	HRV	EEG Band	EEG MPC
(1) Physical museum	Arousal	68.05%	0.68	-	70.93	29.06	36.2	63.79	9/14	1/3	1/1	7/10
**(2) Physical museum + IAPS**	**Arousal**	**71.52%**	**0.72**	**+3.47%**	75.58	24.41	34.48	65.5	10/18	1/2	1/4	8/12
**(3) Physical museum + Em. Rooms**	**Arousal**	**71.52%**	**0.72**	**+3.47%**	79.06	20.93	39.65	60.34	16/18	3/3	3/3	10/12
(1) Physical museum	Valence	74.30%	0.74	-	74.57	25.42	25.88	74.11	9/14	1/3	1/1	7/10
**(2) Physical museum + IAPS**	**Valence**	**77.08%**	**0.76**	**+2.78%**	72.88	27.11	20	80	10/18	2/2	1/4	7/12
(3) Physical museum + Em. Rooms	Valence	76.38%	0.74	+2.08%	69.49	30.5	18.82	81.17	3/18	0/3	0/3	3/12
(1) Virtual museum	Arousal	(TN<60)	-	-	88.07	11.92	44.18	55.81	-	-	-	-
(2) Virtual museum + IAPS	Arousal	71.05%	0.70	**-**	75.22	24.77	39.53	60.46	4/22	0/2	0/3	4/17
**(3) Virtual museum + Em. Rooms**	**Arousal**	**75.00%**	**0.75**	**-**	76.14	23.85	27.9	72.09	1/25	0/4	0/3	1/18
(1) Virtual museum	Valence	67.10%	0.68	-	71.27	28.72	39.65	60.34	4/26	0/3	0/5	4/18
(2) Virtual museum + IAPS	Valence	67.10%	0.68	+0.00%	71.27	28.72	39.65	60.34	3/22	0/2	0/3	3/17
**(3) Virtual museum + Em. Rooms**	**Valence**	**71.05%**	**0.71**	**+3.95%**	74.46	25.53	34.48	65.51	3/25	0/4	0/3	3/18

Level of recognition of arousal and valence in physical/virtual museum exhibition using (1) only physical/virtual museum dataset (2) including IAPS dataset and (3) also including emotional rooms dataset. Average of accuracy in percentage, F-score, increment of accuracy when IAPS and Rooms datasets were included in each case, confusion matrix and features used in each analysis. Bold indicates cases with the highest accuracy.

Concerning the valence recognition in the physical museum, not including IAPS or the emotional rooms data, the accuracy is 74.30%. The best accuracy is obtained by including IAPS data, achieving 77.08%. The confusion matrix is balanced in all cases and features of all analyses were included.

Regarding arousal in the virtual museum, it was not possible to develop a balanced model without including IAPS or emotional room data, because the true negative was below 60%. Including the IAPS data, the accuracy was 71.05%. However, the best accuracy is obtained by including the emotional rooms, achieving 75.00%. Moreover, this model presents a more balance confusion matrix. Both cases only use EEG MPC features.

Concerning valence in the virtual museum, not including IAPS or the emotional rooms, the accuracy is 67.10%. The model including IAPS data presents the same results. The best accuracy includes emotional room data, achieving 71.05% of accuracy. All cases used only EEG MPC features.

### Real vs virtual classification

Table 3 shows the level of recognition of the nature of the stimuli in the museum, classifying if the stimuli are real or virtual. The accuracy is 95.27% and the confusion matrix is balanced. The model uses only one feature of EEG MPC, the first component of the PCA, to achieve this level of recognition.

**Table 3:** Level of recognition of nature of stimuli.

Analysis cases	Feature	Accuracy	F-Score	Confusion matrix				Featured used			
				True high	False high	False low	True low	Total	HRV	EEG Band	EEG MPC
Real vs Virtual	Nature	95.27%	0.95	94.07	5.92	3.47	96.52	1/17	0/3	0/2	1/12

Level of recognition of nature of stimuli (real or virtual), including average of accuracy in percentage, F-score, confusion matrix and features used from each analysis

## Discussion and conclusion

The purpose of this novel and exploratory research was to quantitatively compare psychological and physiological patterns during an emotional experience in a physical environment and their virtualization through a 3D IVE, guiding future emotion elicitation and recognition systems using VR. With this aim in mind, we developed a realistic 3D IVE simulation of an art museum and performed a comparative study involving 60 subjects in a real art museum and its simulation, while they were performing a free exploration of an exhibition. In addition, we included two prior phases including controlled stimuli using 2D pictures and 360° IVEs, in order to study the influence of this data on the accuracy and robustness of the emotional models. The results can be discussed on four levels: i) a comparison of the psychometric scores, ii) a comparison of the physiological patterns, iii) a comparison of the level of emotion recognition and the influence of previously (standardized) controlled stimuli, iv) a comparison of emotional subjective and psychophysiological correlates in VR and real scenarios and its meaning in the framework of the different theories and models of emotion, iv) a methodological assessment and v) limitations of research.

Self-assessment results were used to compare the psychometric patterns. The virtual museum presents slightly more arousal and valence levels than the physical museum. This slight bias could be due to the subjects having no previous VR experience, and the novelty could increase arousal and valence. This should be taken account of in future experiments with these types of subjects. However, only Room 1 presents significant differences in valence considering the usual alpha threshold (α = 0.05). Moreover, considering that this conservative threshold is focused to avoid type I error, we analyse a second threshold (α = 0.1) to decrease the probabilities to perform a type II error and claim incorrectly the null hypothesis of equal means. The vast majority of the stimuli (93.75%) do not present statistically significant differences in self-assessment considering the first alpha threshold (α = 0.05). However, two rooms (1 and 4) present higher arousal, and two rooms (1 and 3) show higher valence in virtual condition considering the second alpha threshold (α = 0.1) [64]. Room 1 presents the biggest differences in the evoked emotion and it could be provoked by a ‘wow’ effect derived also by the novelty and the lack of previous experience in VR. This effect will need to be consider in future research. The results suggest that 3D IVEs are powerful tools for emotional elicitation, since the majority of stimuli do not present significant differences in affective statements reported by the subjects in comparison to those evoked by physical environments, and are appropriate for emotion research. The results also support the use of VR to elicit emotion and are in accordance with previous research [20,30,65], but more confirmatory research is needed in the future, especially considering the new VR devices.

Regarding the physiological pattern comparison, the automatic feature selection of the SVM-RFE algorithm was used. The influence of the features of each analysis on the models could be analysed as the PCA was applied independently for HRV, EEG Band Power and EEG MPC. In emotion recognition of the physical museum, all the models (except for the valence with Emotional Room data) used features of all the analyses to predict mood. Thus, all analyses contributed with information about emotional status. However, the emotion recognition models developed for the virtual museum used only few EEG MPC features. Moreover, the real vs virtual classification model used only the first component of the EEG MPC PCA to discriminate between the real and virtual museum stimuli. These results reveal the important role that brain synchronization plays in the neuro-physiological processes involved in VR, as they can discriminate between virtual and real environments with a level of recognition of over 95% accuracy. In addition, the use of EEG MPC features to recognize emotions in VR suggests that brain synchronization is deeply involved in emotional processes in VR environments. The measures of nonlinear interdependency in EEG have become in the last years an emerging field and they have been applied to analyse perceptual processes, cognitive tasks and disorders [66,67]. Even when these have been applied in virtual reality studies [57,68,69], to our knowledge we present the first evidence of their influence in immersive virtual emotional experiences. In future studies, the correlations between emotions in VR and the synchronization of each brain region should be analysed in depth, since in this exploratory study we use PCA as a feature reduction method in order to perform classification models.

Concerning the level of emotion recognition, we present the first study that develops an emotion recognition system in a 3D IVE, comparing results with the physical environment model. Firstly, we presented the results of the models in the physical and virtual museum without the IAPS or Emotional Rooms dataset, using features extracted from EEG and HRV series gathered from wearable sensors, and properly combined through nonlinear SVM algorithms. The models were validated using LOSO cross-validation, which has been extensively performed in emotion recognition research to validate models [70–72]. The accuracies of the model in the physical museum without IAPS or Emotional Rooms datasets achieve 68.05% in arousal and 74.30% in valence, both balanced in confusion matrix. These results are considerably higher than the level of chance, which is 58% in statistical assessment classification with brain signals (p = 0.05, n>100, 2-classes) [73]. The accuracy of the model in the virtual museum, without including IAPS or Emotional Rooms datasets, is 67.10% in valence and is balanced. However, the model of arousal in the virtual museum does not exceed the balance threshold (>60% of true high and true low), invalidating its accuracy. Therefore, the 3D IVEs show an initial limitation for use in evoking stimuli in emotion recognition systems, especially in arousal recognition.

The emotion stimuli habitually applied in the methodologies of affective computing studies, such as IAPS, include a large number of stimuli to elicit a wide range of emotions with different levels of intensity. This wide range of moods allows the emotion recognition systems to improve their accuracy. However, real-world environment (physical or simulated) stimuli are not created to evoke different ranges of valence and arousal and cannot cover different mood intensity. Thus, the responses to a set of controlled emotional stimuli are included in the emotion models to test if they improve the accuracy of the models. Thus, we analyse the addition of datasets of pre-performed controlled, standardized stimuli which are designed to evoke a range of arousal and valence, including 2D pictures (IAPS) and 360° IVEs (Emotional Rooms).

As can be seen in Table 2, accuracy improves in all models when using IAPS or Emotional Rooms information. Regarding the physical museum, the IAPS and Emotional Rooms datasets provide better accuracy in terms of arousal (71.52%), increasing the accuracy by 3.47% in both cases. The inclusion of IAPS datasets maximizes recognition in terms of valence, achieving 77.08%. Therefore, the inclusion of IAPS works slightly better than the Emotional Rooms phase in physical environments. Regarding the virtual museum, the Emotional Rooms dataset provides better accuracy in terms of arousal (75.00%). In this case, the Emotional Rooms provide 4 points of accuracy more than IAPS and the museum dataset doesn´t achieve a balanced result. The Emotional Rooms dataset also provides better accuracy in terms of valence in the virtual museum (71.05%). The good performance related to the inclusion of the Emotional Rooms dataset in the virtual museum could be because the 360° IVEs provide important information for the recognition of arousal in 3D environments, because both use an HMD. Moreover, the initial accuracy limitation of the model with no previous data is exceeded with the inclusion of the Emotional Room dataset. Thus, a prior phase with 360° IVE controlled stimuli is shown as a powerful methodology to develop emotion recognition models in 3D IVEs. Therefore, the physiological signals allow us to predict the self-assessment in both cases. In future experiments, these results could be optimized using alternative machine learning algorithms and multivariate signal analyses [56] and a confirmatory analysis need to be performed.

Although the arousal and valence self-evaluations were to some extent similar in the virtual and real museums, the two conditions appear to be different in terms of psychophysiological parameters. Moreover, the psychophysiological-based emotional classifiers, virtual and real environments, although they had similar performances with high accuracy, used different features and were affected differently by the introduction of features acquired in stage 1 and 2. Interestingly, the classifier for the virtual environment needs less features than the classifier for the real museum, which suggests that the psychophysiological reaction in the latter was more complex than the former. Our data, therefore, highlights a possible limitation of the application of the circumplex model of emotions to psychophysiological data, since similar subjective experiences (in terms of arousal and valence) did not show unique psychophysiological patterns. For instance, the model does not take in account where the emotion takes place: a VR environment is necessarily unfamiliar and the degree of familiarity does not follow a linear relationship with the similarity to reality (for instance, see [74] for a detailed review of the uncanny valley phenomenon and related issues). Understanding reality in its context is analysed by the Theory of Mind (ToM) and several models suggest that the ToM may modulate emotional perception [75]: even for phobias and their treatment, patients tend to prefer VR because they are cognitively aware that the phobic stimulation is similar but not identical to the real scenario [76]. Recently, an uncanny-valley of the mind reaction was theorized to describe a scenario where VR agents performed in a very similar (but not identical) emphatic way [77]. Similarly, it is possible that being in an environment which is very similar (but not identical) to a real environment will elicit a sense of eeriness. Such a sense of eeriness may interact with psychophysiological responses, but to a lesser extent with the arousal-valence subjective evaluation. It is possible to imagine that, by introducing further dimensions, such as emotional embodiment [78,79] or emotional presence, to the circumplex model of emotion may overcome the current limitations of the model. Regarding emotional presence there are several pieces of evidence that suggest how vividness of emotional experience can affect arousal and valence. For instance, patients with Post Traumatic Stress Disorder (PTSD) report very vivid traumatic emotional memories with high arousal and negative valence. On the contrary, techniques designed to reduce the vividness of such memories also reduce arousal and valence [80]. Finally, our results may also be explained by reference to constructed emotion theory [7]. According to this theory, emotions are predictive and not reactive systems, therefore they depend on what the brain/mind considers the most probable outcome in terms of previous knowledge and sensorial input. Emotional labelling, as we know it, is just an approximation to something similar we have experienced in the past and therefore is not particularly reliable. It is not, therefore, unexpected that VR and real museum experiences are subjectively similar, but different in terms of psychophysiological correlates. Future studies might test the fit of constructed emotion theory to VR data. In this sense, a switch of paradigm may be needed. For instance, as proposed for the psychophysiological correlates of mental disorders [81], we might adopt a data driven approach, based on unsupervised learning algorithms, to identify hidden similarities in psychophysiological reactivity to emotional states.

At a methodological level, the proposed signal processing and machine learning techniques using data from healthcare wearables provide satisfactory levels of recognition, achieving accuracies over 70%. They are presented as a powerful software and hardware equipment to extend the applications of emotion recognition systems including physical real-world environments, and they are in accordance with recent studies using HRV [82] and EEG [83]. However, concerning signal recordings, even when the physical environments allow the analysis of the real impact of one specific environment, these also present the following limitations: i) it is difficult to keep ambient features constant; ii) it is difficult, or even impossible in some cases, to change some environmental features in order to analyse their impact; iii) the extra-cost of developing studies of environments situated far distant and; iv) it is impossible to analyze the impact of an environment before it is constructed. On the other hand, the capacity of virtual simulation to evoke the same emotions as physical environments could be essential in the near future, taking into account the rise of virtuality and the central role that emotion plays in many background processes. Moreover, the capacity of IVEs to be used as stimuli could significantly improve the application of emotion recognition in simulated real-world tasks.

Some possible caveats should be mentioned. This exploratory study aimed at investigating human psycho-physiological patterns of emotions during a free exploration of virtual and real art museums. We used wearable sensors allowing to translate our research to real scenarios, although such sensors a limited number of physiological sensors. In addition, the biosignals could be affected by artefacts especially caused by head movement in the case of virtual museum, and by walking in the case of real museum where we recorded biosignals “in the wild”, i.e. outside of the highly constrained and tightly controlled laboratory paradigms. This is especially true for the EEG series, although many researches have successfully employed such data in combination with HMDs or other wearable devices in naturalistic conditions [13,84,85]. Nonetheless, our results point to the significance of brain synchronization for the emotion recognition in both real and virtual museum scenarios. The psychological self-assessment was performed using retrospective reports, leading to possible bias such as recency, primacy and memory, although our experimental paradigm replicates a real scenario. Note also that the user's emotional perception could be biased by stopping the real or virtual museum exploration. The real and the virtual environments have intrinsic differences in unavoidable physical features such as light, colour and complexity, and these may affect physiological responses. Furthermore, the time of the exploration for each room/piece of art would need to be considered as a confounding/critical factor in future studies because of its possible role in evoking emotions. In particular, it could affect the real vs virtual museum discrimination in case of differences in the time of exploration. In this regards, we recently found that Room 1 and Room 2 of the virtual museum are associated with lower time of visit than the real exhibition [86]. On the other hand, the other 6 stimuli do not show differences in terms of exploration time between real and virtual museums.

This study marks new steps in the discipline of affective computing and its application to environmental physiology and other fields, providing evidence through psychological and physiological comparisons during an emotional experience in real and virtual environments. This exploratory study tries to contribute to overcome passive methods’ limitations of affective elicitation classically used in emotion recognition models, such as pictures, sounds or videos, supporting the use of VR in emotion elicitation. The methodology has implications at commercial and research levels in many disciplines as health, architectural design, urban planning and aesthetics. It could be applied to study the emotional responses of subjects in many specific environments, such as hospitals, schools and factories, where the emotional responses of users play a critical role in daily wellbeing. More specifically, new emotion recognition models will strongly contribute to the development of ambient assisted living, smart environments that change depending on human responses. On the other hand, the new VR set-up allows the analysis of the influence of one parameter, changing it while maintaining the remainder of the environment in a steady state. This will help to develop many studies, impossible to undertake in real environments for physical reasons (e.g. architectural modification of spaces) or security reasons (e.g. phobias therapy). Moreover, it will allow the analysis of environments before their construction, helping in the decision-making process of creating new environments oriented to wellbeing.

## Supporting information

S1 Fig **Comparison between the physical museum (left) and the virtual museum (right).** The photos represent Room 2 and Room 3.
(TIF)

S2 Fig Screenshot of the training environment. (TIF)

S1 File IAPS experimental protocol and physiological signal segmentation and synchronization. (PDF)
