# Supplementary figures and images for: Real vs. immersive-virtual emotional experience: Analysis of psycho-physiological patterns in a free exploration of an art museum
Source: PLoS One. 2019 Oct 15;14(10):e0223881. doi: 10.1371/journal.pone.0223881 (PMC6793875; doi:10.1371/journal.pone.0223881)

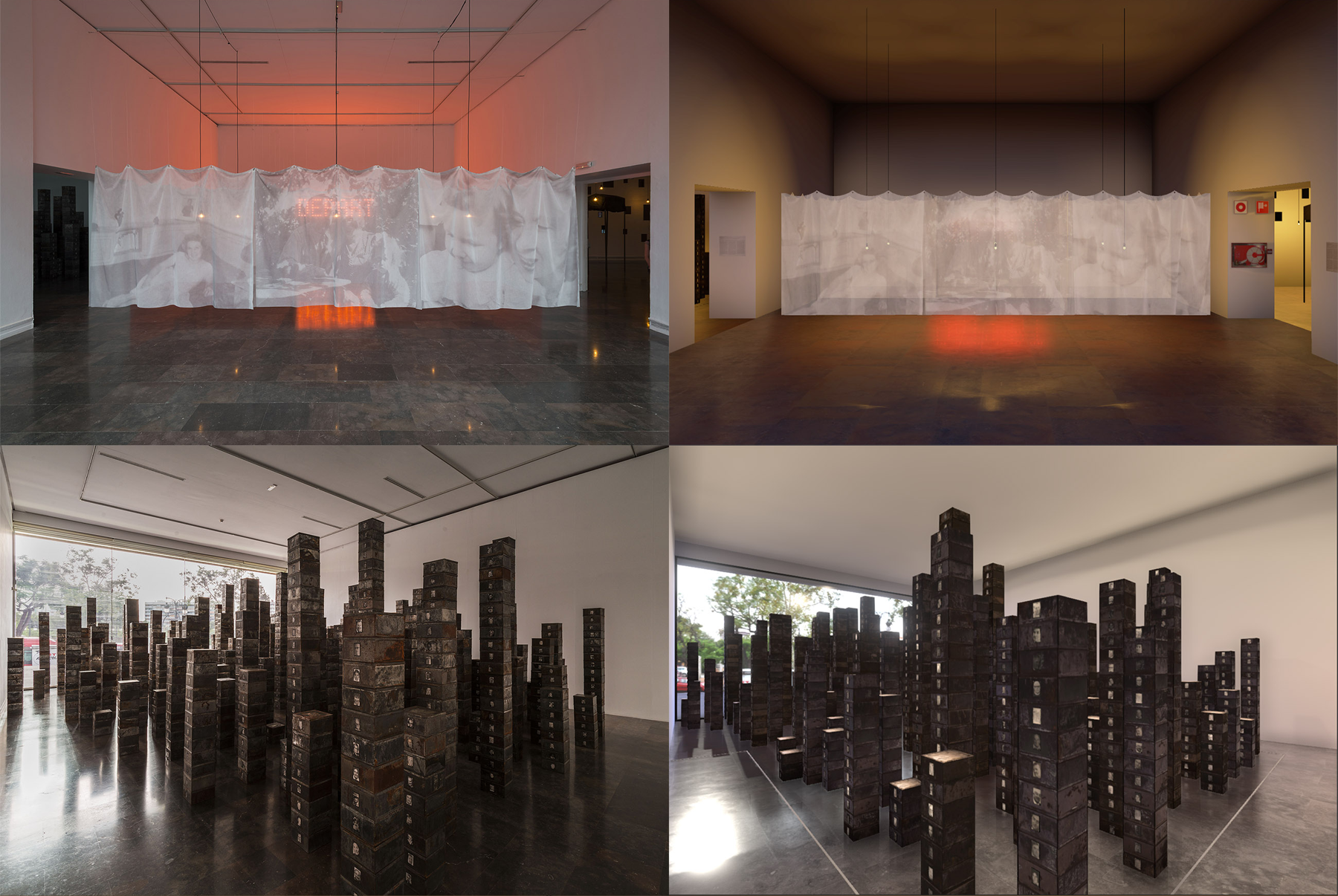

Supplement: S1 Fig — Comparison between the physical museum (left) and the virtual museum (right). The photos represent Room 2 and Room 3. (TIF) [file pone.0223881.s001.tif]

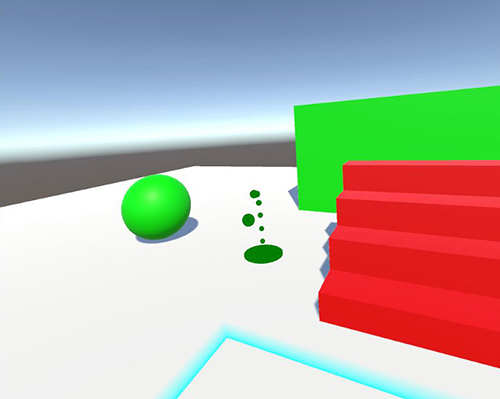

Supplement: S2 Fig — (TIF) [file pone.0223881.s002.tif]
