# Supplementary material for: Real vs. immersive-virtual emotional experience: Analysis of psycho-physiological patterns in a free exploration of an art museum
Source: PLoS One. 2019 Oct 15;14(10):e0223881. doi: 10.1371/journal.pone.0223881 (PMC6793875; doi:10.1371/journal.pone.0223881)
Supplement: S1 File — (PDF) [file pone.0223881.s003.pdf]

Real vs. immersive-virtual emotional experience: Exploiting psycho-physiological patterns in a free exploration of an art museum

Supporting information

IAPS experimental protocol

Table 1 shows the rating of the images used in each session of the experimental protocol of Stage 1.

Table 1. Rating of IAPS images used in Stage 1

**Arousal level**	**Valence level**	**N pics.**	**Valence rating**	**Valence range**	**Arousal rating**	**Arousal range**
N	N	6	4.99 ± 0.14	4.82 ÷ 5.21	2.80 ± 0.26	2.35 ÷ 3.03
A1	V1	7	4.26 ± 0.33	3.71 ÷ 4.75	3.59 ± 0.22	3.31 ÷ 3.88
A1	V2	6	5.41 ± 1.07	4.38 ÷ 6.50	3.67 ± 0.17	3.53 ÷ 3.95
A1	V3	7	7.56 ± 0.51	6.81 ÷ 8.11	3.58 ± 0.28	3.20 ÷ 3.92
A2	V1	7	3.06 ± 0.71	2.09 ÷ 4.02	4.71 ± 0.22	4.28 ÷ 4.97
A2	V2	6	5.21 ± 1.20	4.09 ÷ 6.40	4.62 ± 0.31	4.20 ÷ 4.94
A2	V3	7	7.70 ± 0.70	6.45 ÷ 8.30	4.45 ± 0.22	4.07 ÷ 4.62
A3	V1	7	2.34 ± 0.58	1.84 ÷ 3.17	5.94 ± 0.22	5.65 ÷ 6.20
A3	V2	6	4.80 ± 1.52	3.27 ÷ 6.31	5.54 ± 0.19	5.35 ÷ 5.90
A3	V3	7	7.57 ± 0.60	6.53 ÷ 8.06	5.69 ± 0.26	5.27 ÷ 5.96
A4	V1	7	2.01 ± 0.55	1.29 ÷ 2.70	6.61 ± 0.26	6.29 ÷ 6.94
A4	V2	6	4.90 ± 1.71	2.96 ÷ 6.62	6.78 ± 0.16	6.55 ÷ 6.99
A4	V3	7	7.44 ± 0.33	6.87 ÷ 7.88	6.81 ± 0.53	6.23 ÷ 7.39

Physiological signal segmentation and synchronization

1) Previous controlled stimuli

In Stage 1, the IAPS images and the physiological signal recorded were synchronized using the software iMotions (iMotions A/S, Denmark). The segmentation of IAPS is explained in the protocol. In order to use the same number of blocks as in the emotional rooms, we used, for the classification model, only the four outermost blocks, combining low arousal (A1), high arousal (A4), negative valence (V1) and positive valence (V3). Each block was considered as an independent stimulus and had a duration of 70 seconds.

Regarding Stage 2, the software allowed the insertion of live markers in the physiological signal recording. The researcher inserted a live marker when each room began to display in the Samsung Gear HMD, so each room (stimuli) was associated with a specific live marker. Then, each time window of each stimulus was segmented using these markers. In conclusion, in the IAPS and Emotional room stages, a subject had four stimuli and each was theoretically situated in one quadrant of the CMA, providing a controlled stimulus set that included all arousal and valence combinations.

2) Physical museum exhibition

The physiological signals were recorded using iMotions software (iMotions A/S, Denmark), running in the laptop carried by the subjects. In order to record the positions of the subjects, we used a GoPro camera Fig. 1. To synchronize the video with the physiological signals, we needed a synchronization point. When the signal and video recording started, the camera was focused onto the laptop and the researcher inserted a live marker software. By using this marker in the signals, and the frame of the video

where the researcher inserted it, we could synchronize the video and the physiological signals.

To obtain the data about the navigation of the subjects from the video, we designed a tool using Microsoft Virtual Studio in C++ language. The software simultaneously showed two items: a video of the recorded exploration of the exhibition and a plan of the exhibition. The device includes two buttons to advance and rewind the video with 1-second jumps. In addition, it allows us to enter the position of the subjects in each frame of the plan through "clicks", using the video as a reference. The researcher watched the videos and positioned the subjects in the plan at one second intervals. Finally, the navigation was saved in a file with the route sampled every second. The timeline of the video was synchronized with the physiological signal timeline using the previously inserted live marker.

8 stimuli were defined, 5 when the subjects visited each room and 3 when they viewed the pieces of art. A visit to an area starts when a subject enters the area and finishes when the subject leaves the area. The area of each room was defined by its walls and the area of each piece of art was defined by an area of influence, shown at Fig. 5. If an area has only one visit, the stimuli are defined by the time taken to visit the area. If the subject makes more than one visit to an area and the time between the visits is less than 15 seconds, the visits were merged and considered as a single visit. After this pre-process, if there were more than one visit to the same room/stopping point, the stimuli were defined by the longest visit. In addition, a visit needed to have a duration of at least 40 seconds to be considered as a valid stimulus.

3) Virtual museum exhibition

The physiological signals were recorded using iMotions software. A script in Unity was developed and inserted into the scenario with 2 tasks: (1) recording the position of the subject in each frame of the virtual environment and exporting it in a csv; (2) sending a live marker automatically to the iMotions software when the scenario and the recording of the position started, allowing synchronization between the navigation and the physical signal.

The navigation recording was resampled to the same frequency as the physical environment navigation (1 Hz)**.** Following this, applying the same methodology, we defined the stimuli in exactly the same way as for the real museum.

4) *Real vs. Virtual* Classification

Regarding the real-virtual classification, a dataset was created concatenating the features of the stimuli in the physical museum (Stage 3.1) and the virtual museum (Stage 3.2), mixing virtual and physical stimuli in the same dataset. The output of this dataset is the nature of the stimulus (real or virtual). Thus, the pattern recognition classifier algorithm tries to recognize if the stimuli are virtual or real, by seeking to analyse which features enable the classifier to determine the nature of the stimuli.
